# A Comparison of the Immunostimulatory Effects of Polysaccharides from Tetraploid and Diploid* Echinacea purpurea*

**DOI:** 10.1155/2018/8628531

**Published:** 2018-07-09

**Authors:** Guang Yang, Keke Li, Cui Liu, Peipei Peng, Mei Bai, Jiaqi Sun, Qingling Li, Zhuohong Yang, Yuesheng Yang, Hong Wu

**Affiliations:** ^1^State Key Laboratory for Conservation and Utilization of Subtropical Agro-Bioresources, South China Agricultural University, Guangzhou 510642, China; ^2^Guangdong Technology Research Center for Traditional Chinese Veterinary Medicine and Natural Medicine, South China Agricultural University, Guangzhou 510642, China

## Abstract

Polyploidization is an effective means of improving the active components and quality of secondary metabolism in medicinal plants. In the present study, we compared the immunostimulatory effects of crude polysaccharides from tetraploid and diploid* Echinacea purpurea*. The results showed that the carbohydrate contents of crude polysaccharide of tetraploid* E. purpurea* (CPE4) and diploid* E. purpurea* (CPE2) were 85.51% and 44.65%, respectively. ^1^H-nuclear magnetic resonance (NMR) spectroscopy and gel-permeation chromatography (GPC) analyses showed no major differences in the overall structure and molecular weight of polysaccharides between CPE4 and CPE2. However, some differences in the relative content of the same polysaccharides group were observed between CPE4 and CPE2. In in vitro tests, EP4 could stimulate lymphocyte proliferation and secretion of cytokines maximally at the concentration of 0.0312 mg/mL, and EP2 could stimulate lymphocyte proliferation and secretion of cytokines maximally at the concentration of 0.125 mg/mL. In in vivo tests, EP4 was more effective at promoting the proliferation of lymphocytes and secretion of cytokines in mice immunosuppressed by cyclophosphamide than EP2 at the same concentration. Taken together, these data demonstrated that the relative content of the partial polysaccharides group is increased, and the immunoregulatory effect is enhanced in tetraploid* E. purpurea*.

## 1. Introduction

*Echinacea purpurea* (*E. purpurea*) Moench is a type of* Echinacea* plant (Asteraceae).* E. purpurea*, popularly known as purple coneflower, is an important medicinal plant native to North America. Extracts from the plant show antioxidative, antibacterial, antiviral, and antifungal properties and are thus commonly used for treating common cold, respiratory, and urinary diseases [1]. Recently, the antitussive and bronchodilatory effects of* Echinacea* were revealed by pharmacodynamic studies [2]. The potential active compounds in* E. purpurea* include polysaccharides, glycoproteins, alkylamides, caffeic acid derivatives, volatile oils, and alkaloids. The polysaccharides, glycoproteins, alkylamides, and caffeic acid derivatives have shown immunostimulatory properties [3–7].

Polysaccharide is a main active component in natural medicinal plants with many bioactivities, such as immunomodulation, antiviral activities, and antiproliferative activity [8].* Cordyceps* polysaccharide can greatly improve the immune function of the Chicken Newcastle Disease vaccine [9].* Atractylodes* polysaccharide and* Pachymaran* and* Astragalus* polysaccharide strengthen immunity and have antibacterial, antivirus, antitumor, and antiparasitic effects [10–14]. Recent studies have focused on the immunomodulatory effects of* Echinacea* [15], and* Echinacea* polysaccharide may enhance the production and secretion of macrophage TNF-*α*, IFN-*γ*, IFN-*β*, IL-2, and IL-6 [16, 17].* Echinacea* contains polysaccharides of various molecular weights; among them,* Echinacea* arabinose regulates the macrophage secretion of interferon [6], and the fructan polysaccharides of* Echinacea* have potential biological effects on cancer [18].

Polyploidy is frequently accompanied by conspicuous changes in morphology and metabolite production. In particular, polyploidy may increase the content of active pharmaceutical ingredients in medicinal plants [19, 20]. For example, the increased size and alkaloid contents suggest that tetraploid seeds contain better alkaloids than diploid seeds in* Datura* [21]. Additionally, the main active pharmaceutical ingredients of the genus* Scutellaria* were substantially higher in autopolyploids and allopolyploids than in haploids [22], and, compared with the parent explants, the level of* Lycium barbarum* polysaccharide is higher in tetraploid than in diploid plants [23]. Previous studies have shown the total fresh root weight and total dry root weight of tetraploid* Echinacea* to be higher than those of diploid plants, and tetraploid plants have higher contents of caffeic acid derivatives and alkamides than diploid* E. purpurea* plants [20]. The content of bioactive secondary metabolites in most polyploid medicinal plants is higher than that in diploid plants; therefore, the induction of polyploidy will certainly result in an increase of biological activity [24, 25].* Echinacea* polysaccharides have significant effects on the immune system [4]; however, the effects of* Echinacea* ploidy levels on polysaccharide content and immune modulation remain unknown. In the present study, ploidy manipulation was used to obtain tetraploid* E. purpurea*. We used modern pharmacology methods to study the effects of ploidy level on the content and immune activity of polysaccharides in vitro and in vivo. The present study provides a theoretical basis for breeding through induction of polyploidy to achieve a higher yield of biomass and bioactive compounds to increase the clinical treatment effects of* E. purpurea*.

## 2. Materials and Methods

### 2.1. Materials and Reagents

Primary* E. purpurea* seeds were supplied by the Company of Plantation Products (Norton, MA, USA) and cultivated in the Garden of Chinese Medicinal Plants on the campus of South China Agricultural University. The offspring seeds were used in the present study. Tetraploid* E. purpurea* were obtained from Dr. Wu's Lab (Guangdong Technology Research Center for Traditional Chinese Veterinary Medicine and Natural Medicine, South China Agricultural University). ConA and MTT were purchased from Sigma-Aldrich of China. Roswell Park, Memorial Institute (RPMI)-1640 medium, phosphate-buffered saline (PBS), and pen/strep were purchased from GIBCO, fetal bovine serum (FBS) was purchased from BOVOGEN, and ELISA assays to detect IL-2, IFN-*γ*, and TNF-*ɑ* were purchased from Shanghai Meilian Biological Technology.

### 2.2. Chromosome Counting Method of* E. purpurea*

 The* Echinacea* root tips of about 10 mm were dissected and washed with pure water. The root tips were socked for 3-4 h in 0.05 % (w/v) colchicine water solution under 4°C and washed with running tap water for about 15 min, left to steep in water for 8 min, and then put in Carnoy's solution containing acetic acid and 95% ethanol in a ratio of 1:3 (v/v) for at least 24 h at 4°C for fixation. The fixed root tips were washed again with running tap water for about 15 min, left to steep in water for 8 min, and hydrolyzed in 1 M HCl for 5 min at 60°C. After hydrolysis, the root tips were washed again with running tap water for 15 min and left to steep in water for 8 min. Subsequently, these root tips were stained with 20% (v/v) carbolfuchsin solution for 2 min, squashed on slides under a cover glass, and observed under a microscope for the selection of images of well-spread metaphase chromosomes.

### 2.3. Preparation of Crude Polysaccharides from* E. purpurea*

Hot water extraction, followed by ethanol precipitation, was used for the production of crude polysaccharides from tetraploid (CPE4) and diploid (CPE2)* E. purpurea*. First, samples (300 g) of different ploidy dried whole-plant powder of* E. purpurea* were treated with petroleum ether at 80°C and refluenced for 2 hours to remove lipophilic substances. Subsequently, 2400 mL of water was added to the remaining lipophilic substance-free sample [sample to water ratio, 1:8 (v/v)], followed by boiling. The supernatant was collected, and this process was repeated three times, once an hour. After filtering using gauze, the mixed decoction was concentrated to 300 mL, and pure alcohol (99%) was added to the mixture to obtain a final alcohol concentration of 80% (v/v). After decoction at 4°C overnight, followed by laying for 18 h, which was repeated three times, the invalid portion was discarded, and the deposition was collected and freeze-dried (Eppendorf AG-5805). The crude polysaccharide samples were obtained from two ploidy* E. purpurea*. The weights of CPE2 and CPE4 were 17.46 and 22.46 g in the 300 g samples of diploid and tetraploid* E. purpurea*, respectively. The extraction yield was calculated as the weight ratio of CPE and the dried* E. purpurea* sample. The extraction yield of CPE2 and CPE4 was 5.82% and 7.82%, respectively. Subsequently, 5 mg/mL CPE2 and CPE4 was used to determine the carbohydrate contents in CPE using the phenol-sulfuric acid method in three replications [25, 26]. The regression equation of the glucose standard was used: A = 0.00246C + 0.0343, R2 = 0.99924 (*n* = 5), where A is the OD490 of the sample, C is the concentration of glucose in the sample solution, and R2 is the coefficient of determination.

### 2.4. ^1^H-Nuclear Magnetic Resonance (NMR) Spectroscopy

The diploid and tetraploid* E. purpurea* crude polysaccharides were dissolved in deuterium oxide (D2O) (adding acetone as internal standard, sample concentration ≥10 mg/mL) in NMR glass tubes, and the supernatants were scanned using Bruker AVANCE 600. The following NMR conditions were used: 5 mm BBO probe; temperature measurement, 297 K; frequency, 600.13 MHz; spectrum width (SW), 9600 Hz; TD, 38460; the pulse width (pw), 12.34 *μ*s; acquisition time, 4 s; pulse relaxation, 5 s; acquisition frequency, 128; pulse angle, 90; and gain value, 181.

### 2.5. Gel-Permeation Chromatography (GPC)

The analyses were performed on an SHIMADZU LC-20AT GPC system equipped with a RID-10A detector. Three columns (TOSOH Company) of TSKgel G-3000PW_XL_, TSKgel G-5000PW_XL_, and TSKgel G-6000PW_XL_ were in series with each other. The eluent was 20 mM Na_2_HPO_4_-NaH_2_PO_4_, (pH = 7). Freeze-dried diploid and tetraploid crude polysaccharides were dissolved in eluent (1-10 mg/mL) and filtered (0.45 *μ*m). The flow rate was 0.5 mL/min, and the analysis time was 80 min. MW 4.32×103, 1.26×104, 6.06×104, 1.10×105, and 2.89×105 glucans (National Institute of Metrology, China) were the molecular weight standards.

### 2.6. Animals

Kunming specific pathogen-free (SPF) mice, weighing 20±4 g, at equal proportions of males and females, were purchased from the Guangdong Medical Laboratory Animal Center, and the mice were housed individually in a windowless room with controlled temperature and light (24±2°C, humidity 50%-70%, and light cycle of 08:00-20:00). After acclimation for one week, the mice were maintained with free access to standard laboratory pellet diet and water. All procedures involving animals throughout the experiments were conducted in strict accordance with the Chinese legislation on the use and care of laboratory animals. Moreover, all efforts were made to minimize suffering.

### 2.7. Determination of Tests In Vitro

Five-week-old female and male SPF mice (20±4 g, purchased from the Guangdong Medical Laboratory Animal Center, approval no. SYXK 2014-0136) were housed individually in a windowless room with controlled temperature and light. After acclimation for one week, the mice were provided free access to experimental diet. At the end of the experiment, the mice were sacrificed under anesthesia, the spleen was separated and steeped in 75% alcohol for 5 min, and the spleen lymphocytes isolated were cultured in FBS/RPMI1640 medium. For testing in vitro, first, CPE4 and CPE2 solutions of 1.0 mg/mL were prepared with deionized water, and subsequently the solutions were diluted into eight working concentrations (0.5–0.0039 mg/mL) in twofold serial dilutions with RPMI-1640. The spleen lymphocytes were adjusted to 2×10^6^ cells/mL in 96-well microtiter plates, and CPE4 and CPE2 were added at a final volume of 200 *µ*L. The cells were incubated at 37°C and 5% CO_2_ for 48 h in the absence or presence of 10 *µ*g/mL concanavalin A (ConA) [27–29]. The transformation capacity of lymphocytes was evaluated using the MTT assay for T-cell stimulation assay, and the concentrations of IL-2 and IFN-*γ* were measured by ELISA according to the manufacturer's instructions.

### 2.8. Determination of Tests In Vivo

The effect of crude polysaccharide of ploidy* E. purpurea* on the immune function of Jimpy mice induced with cyclophosphamide was studied empirically [30]. Five-week-old female and male SPF mice (20±4 g, purchased from the Guangdong Medical Laboratory Animal Center, approval no. SYXK 2014-0136) were housed individually in a windowless room with controlled temperature and light. The mice were randomly divided into 9 groups, with 10 mice in each group, as follows: the normal control group (C); the model control group (M); the Esberitox group (positive control E); the diploid* E. purpurea* groups (low, medium, and high doses); and the tetraploid* E. purpurea* groups (low, medium, and high doses). The injection was performed after a one-week acclimation. The dose of injection for each mouse was 20 mL/kg BW. First, the mouse model was established by injecting cyclophosphamide intraperitoneally for 4 days; the normal control group did not receive any drug treatment. Subsequently, the drugs were administered to the corresponding test groups, and the normal control group and model control group were intragastrically administered with saline once a day, continuing for six days. The drugs of all experimental groups were prepared with 20 mL of either saline solution, which contained 80 mg/kg BW cyclophosphamide, 90 mg/kg BW Esberitox, 40, 80, and 160 mg/kg BW CPE2, and 40, 80, and 160 mg/kg BW CPE4.

At 24 h after the last administration, all mice were sacrificed under anesthesia. The spleen lymphocytes isolated from each test group were cultured in FBS/RPMI1640 medium. The spleen lymphocytes were adjusted to 2×10^6^ cells/mL in 96-well microtiter plates at a final volume of 100 *µ*L. The cells were incubated at 37°C and 5% CO_2_ for 48 h in the absence or presence of 10 *µ*g/ml ConA. The lymphocyte transformation capacity was tested using the MTT assay for T-cell stimulation, and the concentrations of IL-2, IFN-*γ*, and TNF-*α* were measured using ELISA according to the manufacturer's instructions for the IL-2 and IFN-*γ* and TNF-*α* cytokine secretion assays.

To measure the viscera indices, five-week-old female and male SPF mice were randomly divided into 9 groups, and the mouse model was established by injecting cyclophosphamide intraperitoneally at 80 mg/kg BW for 4 days; the normal control group did not receive any drug treatment. Subsequently, all groups were intragastrically administered at 20 mL/kg BW once a day for 6 successive days; specifically, saline was used as normal control group and model control group, the Esberitox (90 mg/kg BW) was used as E group, and the CPE (at 40, 80, and 160 mg/kg BW) was used as CPE-L, CPE-M, and CPE-H, (CPE2 and CPE4), respectively. At 24 h after the last administration, all mice were sacrificed under anesthesia, their body weights and weights of the heart, liver, spleen, lungs, and kidneys were recorded, and the organ indices were calculated.

### 2.9. Statistical Analyses

The data are expressed as the mean ± SD and evaluated using one-way analysis of variance, followed by Duncan's multiple range tests with the software SPSS 20.0. Significant differences were considered at* p* < 0.05, and extremely significant differences were considered at* p* < 0.01.

## 3. Results

### 3.1. Polyploid Level Determination of* E. purpurea*

**Figure 1:**
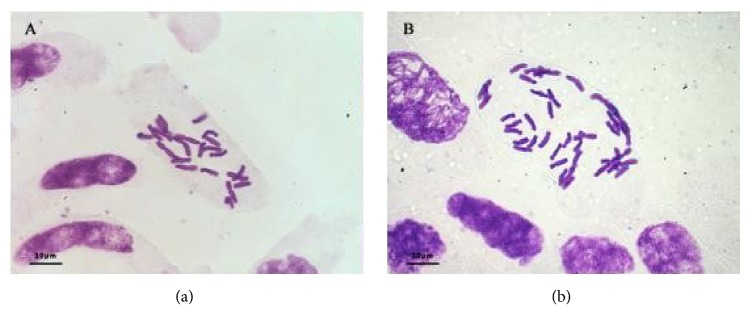
Chromosome numbers of tetraploid and diploid* E. purpurea*: (a) chromosomes in tip cells of diploid* E. purpurea* (2x = 22); (b) chromosomes in tip cells of tetraploid* E. purpurea* (4x = 44).

The determination of ploidy was based on observations of chromosome number. The detailed procedures were described previously [26]. Plants with all root tip cells showing 2x=22 chromosomes were determined to be diploid, and those with all root tip cells showing 2x=44 chromosomes were determined to be tetraploid. The chromosome numbers of tetraploid and diploid* E. purpurea* are illustrated in Figure 1. These observations demonstrated the successful establishment of tetraploid* E. purpurea*.

### 3.2. Determination of Crude Polysaccharide Carbohydrates

Standard curves and equations were established to calculate the carbohydrate content of CPE2 and CPE4. Physicomechanical values were calculated as follows: E(%) = [(5/V)×C]/0.01×106, where V stands for the volume of sample solution, C is the concentration of glucose in the sample solution, and 0.01 is a conversion factor. The results are listed in Table 1. The results showed that the carbohydrate contents of CPE2 and CPE4 were 44.65% and 85.51%, respectively.

### 3.3. ^1^H-NMR Spectroscopy Analysis

**Figure 2:**
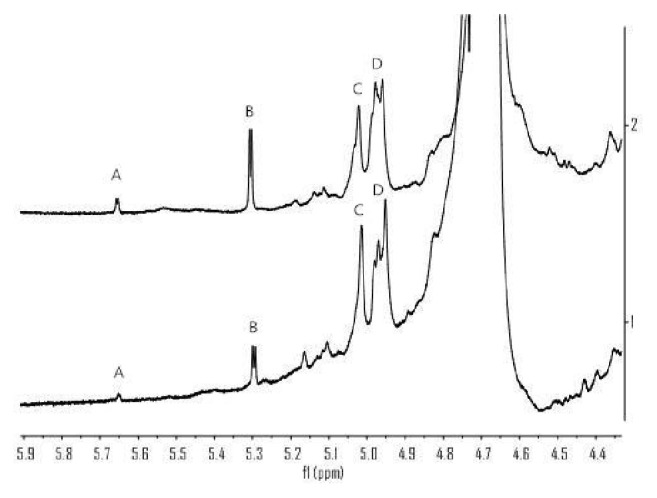
^1^H-NMR spectra of tetraploid and diploid* E. purpurea* polysaccharide: (1) diploid* E. purpurea* polysaccharide; (2) tetraploid* E. purpurea* polysaccharide.

To compare the characteristics of the overall structure of diploid and tetraploid* E. purpurea* crude polysaccharides, ^1^H-NMR spectroscopy was employed. The ^1^H-NMR spectra revealed that the chemical shift values of the polysaccharide anomeric protons are always located at low field in the range from 4.4 to 5.8 ppm. Generally, the proton signal number of polysaccharide anomeric protons in the above field determined the number of monosaccharide types [31]. The ^1^H-NMR spectra of diploid and tetraploid* E. purpurea* polysaccharides are shown in Figure 2. The ^1^H-NMR spectrum of tetraploid polysaccharide had four main proton signals at the chemical shift values of 5.64, 5.30, 5.02, and 4.96 ppm, which were consistent with the proton signals of ^1^H-NMR spectrum of diploid polysaccharide at the chemical shift values of 4.4 to 5.8 ppm. The result illustrates that diploid polysaccharide and tetraploid polysaccharide have the same monosaccharide type, composition, and linkage. According to the rough estimation of the peak height of polysaccharides groups with the same chemical shift values, the relative content of A and B polysaccharides groups was higher in tetraploids than in diploids. In contrast, the content of C and D polysaccharide groups was lower in tetraploids than in diploids.

### 3.4. GPC Analysis

**Figure 3:**
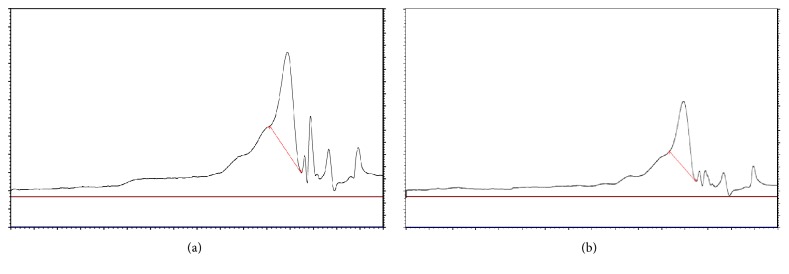
GPC spectra of tetraploid and diploid* E. purpurea* polysaccharide: (a) diploid* E. purpurea* polysaccharide; (b) tetraploid* E. purpurea* polysaccharide.

To compare the differences in the molecular weights of diploid and tetraploid* E. purpurea* crude polysaccharides, GPC was employed. The GPC chromatograms of diploid and tetraploid* E. purpurea* polysaccharides are shown in Figure 3. The peaks from the GPC spectra of polysaccharides appear before the retention time (tR) of 62.5 min as the result of glucans molecular standards (data not shown). As shown in Figure 3, the GPC peaks of the tetraploid and diploid* E. purpurea* polysaccharides were almost the same, which indicated that the tetraploid and diploid* E. purpurea* polysaccharides had the same molecular weight. The main polysaccharides of the tetraploid and diploid* E. purpurea* had the same molecular weight of 5 kDa at the tR of 59.666 min and 59.412 min, respectively. Additionally, both of the tetraploid and diploid* E. purpurea* had polysaccharides eluted from tR of 45 min to tR of 57 min with the molecular weights from 300 to 10 kDa. Based on the above results, there were no new molecular weight polysaccharides in the tetraploid* E. purpurea* compared with the diploid* E. purpurea*.

### 3.5. Change of Lymphocyte Proliferation In Vitro Test

Alterations in lymphocyte proliferation were determined using MTT experiments. The A567 values of every group are listed in Table 2. The A567 values in the CPE2 and CPE4 at 0.0039-0.5 mg/mL group were significantly larger than those of the corresponding ConA control group (*p* < 0.05); CPE2 (SI = 1.42) at 0.125 mg/mL, and CPE4 (SI = 1.409) at 0.0312 mg/mL group presented the highest stimulation index (SI) of lymphocyte transformation.

### 3.6. Effect of Polysaccharides of* E. purpurea* on Cytokine Response

The IL-2 and IFN-*γ* contents of the CPE2 and CPE4 groups are illustrated in Table 3. The IL-2 and IFN-*γ* contents of the CPE2 and CPE4 at 0.0039 mg/mL-0.5 mg/mL were significantly larger than that of the corresponding ConA control group (*p* < 0.05), and the degrees of changing tendency from CPE2 and CPE4 were similar. The CPE2 (879.48 pg/mL) at 0.125 mg/mL and CPE4 (866.99 pg/mL) at 0.0312 mg/mL groups presented the highest secretion of IL-2 in lymphocytes. The CPE2 (1115.04 pg/mL) at 0.125 mg/mL and CPE4 (1430.00 pg/mL) at 0.0312 mg/mL presented the highest secretion of IFN-*γ* in lymphocytes. At every time point, the IFN-*γ* content in the CPE4 at 0.0312 mg/mL was significantly higher than that in the CPE2 groups (*p* < 0.05).

### 3.7. Lymphocyte Proliferation In Vivo

**Figure 4:**
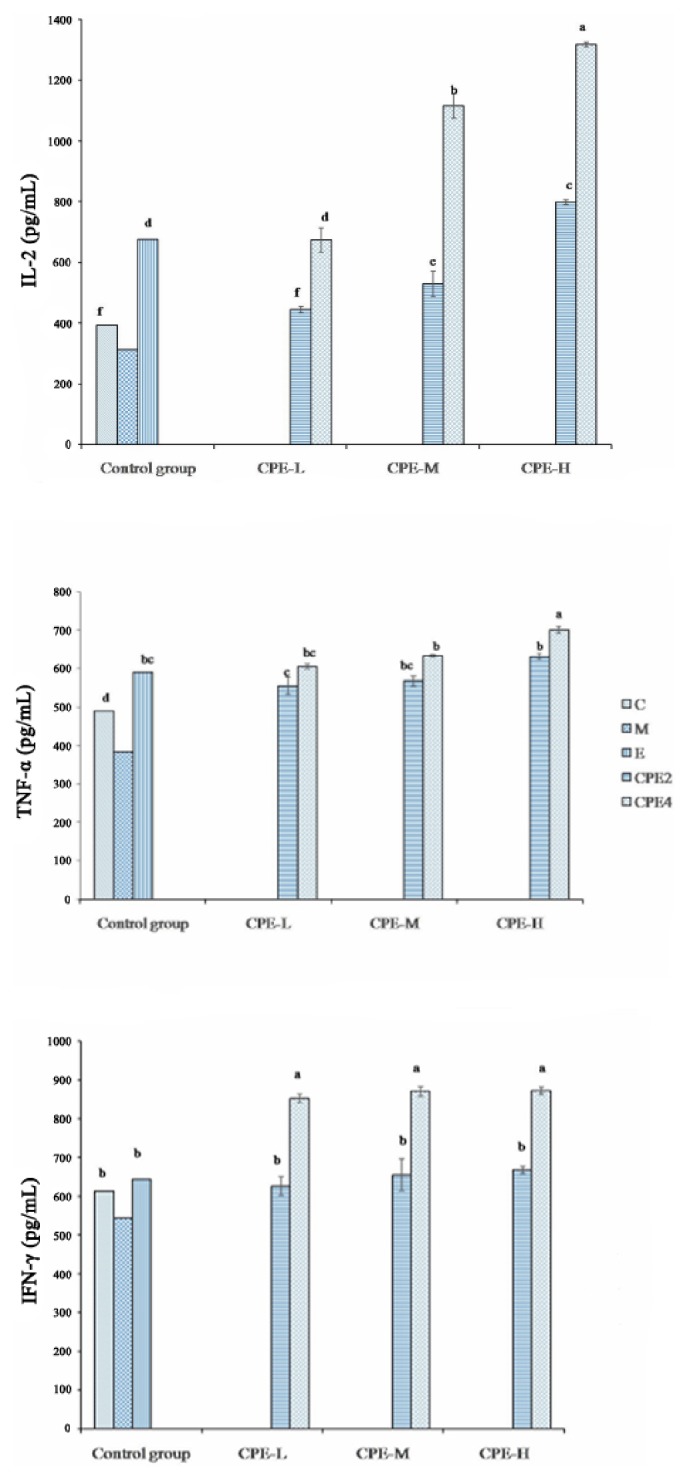
Effects of tetraploid and diploid* E. purpurea *on the production of IL-2, TNF-*α*, and IFN-*γ* in mice immunosuppressed by cyclophosphamide.

The role of the activated CPE2 and CPE4 in lymphocyte proliferation and cytokine secretion was investigated. As shown in Table 4, CPE2 and CPE4 enhanced the proliferation of lymphocytes of mice inhibited by cyclophosphamide significantly more than the M group (*p* < 0.05). The results showed that the effect of CPE4-H was most evident (SI = 2.112), and at the same concentration, CPE4-H was higher than the CPE2 groups in that proliferation of lymphocytes in mice was inhibited by cyclophosphamide, but there was no significant difference between the CPE2-H and S groups. As shown in Figure 4, the IL-2 (pg/mL), IFN-*γ* (pg/mL), and TNF-*α* (pg/mL) contents of the CPE2 and CPE4 groups were significantly larger than those of the C and M groups (*p* < 0.05). The results showed that CPE4-H could significantly induce lymphocytes to secrete IL-2, IFN-*γ*, and TNF-*α*, and the related contents were 700.814 pg/mL, 871.826 pg/mL, and 1317.644 pg/mL, respectively. The IL-2, IFN-*γ*, and TNF-*α* contents of the CPE4-H group were significantly greater than those of the CPE2 and E groups, especially compared with the CPE2 group with regard to IL-2 secretion (*p* < 0.05).

### 3.8. Change of Viscera Indices of Mice

The effects of CPE2 and CPE4 on the weight index of important visceral organs of mice immunosuppressed by cyclophosphamide are compared in Table 5. The viscera indices of the heart, liver, spleen, lungs, and kidneys were significantly lower for the M group than for the C group, and cyclophosphamide could reduce the immune organ indices of mice. With the increase of CPE2 and CPE4 concentration, the viscera indices increased. In CPE4-H, which has the highest count on the viscera indices of the heart, liver, and spleen for all groups, the viscera indices were 0.957, 5.745, 1.125, 0.874, and 1.636 mg/g, respectively. The viscera indices of group CPE4 were significantly larger than that of group CPE2 at the same concentrations. In addition, group E as a positive control could increase the viscera indices of mice immunosuppressed by cyclophosphamide (*p* < 0.05).

## 4. Discussion

Chromosome doubling in medicinal plants typically leads to changes of shape, structure, and secondary metabolites and usually also to a higher content of medicinal ingredients [24, 29, 30, 32–35]. In* E. purpurea*, polysaccharides, caffeic acid and its derivatives, and alkylamides have certain immunomodulatory activities [4–6]. Previous studies have shown that the contents of cichoric acid and alkylamides in the root of* E. purpurea* were higher in tetraploids than in diploids [26]. The present study also demonstrated that the accumulation of polysaccharides in the whole herb of tetraploid* E. purpurea* was significantly higher than that in diploids. Similar studies have also shown that the content of water-soluble polysaccharides in tetraploid wolfberry fruit is significantly higher than that in diploids [23]. These studies indicated that chromosome doubling is beneficial to the accumulation of polysaccharides in plants. Regarding the impact of ploidy changes on plant metabolites, many researchers have confirmed through studies of genomics and proteomics that the cause of the change is that the chromosome doubling changes genome expression.

In the present study, there was an increased accumulation of* E. purpurea* polysaccharides through chromosome doubling, but the results of ^1^H-NMR spectra and GPC chromatogram showed that there were no differences between the diploid and tetraploid polysaccharides in the overall structure and molecular weight. The results indicated that the chromosome doubling did not lead to the synthesis of new* E. purpurea* polysaccharides but increased the amount of the original accumulation of partial polysaccharides group. The composition of plant polysaccharides is complex; researchers have isolated an acidic arabinogalactan (70 kDa) and an arabinogalactan-protein (1200 kDa) from* E. purpurea* herb-pressed juice [36]. The 4-o-methyl glucuronic acid polysaccharide (35 kD, PSI) and acidic rhamnosus arabinogalactan (45 kD, PSII) have been isolated from the* E. purpurea* aerial parts aqueous extract. From* E. purpurea* stems and leaves, researchers have obtained a xyloglucan (79.5 kD) and purified polysaccharide (450 kD) [35–39]. The metabolism mechanism of the chromosome doubling to change medicinal plant secondary metabolites is not clear [34]. The result may reflect the fact that the genes that regulated the biosynthesis of polysaccharides in tetraploid plants could be dose-dependent. Future studies of the variation in the mRNA levels of the genes encoding polysaccharides synthetase in* E. purpurea* with different ploidy levels will provide additional evidence to elucidate the regulatory mechanism through which the ploidy level impacts the content of metabolites in* E. purpurea*.

The diploid* E. purpurea *polysaccharides have certain immune activity [5, 33–35]. Through comparing the differences of tetraploid and diploid* E. purpurea* in immune activity, we showed that the chromosome doubling significantly increased immune activity. The in vitro experiments showed that the tetraploid and diploid* E. purpurea* polysaccharides at concentrations of 0.0039 mg/mL-0.5 mg/mL could promote the proliferation of normal mouse spleen lymphocytes stimulated by ConA. Under the same conditions, the tetraploid* E. purpurea* polysaccharides at low concentrations (0.0312 mg/mL) maximally stimulated lymphocyte proliferation, while mouse spleen lymphocyte stimulation was optimal when the diploid* Echinacea* polysaccharide concentration was 0.125 mg/mL. However, tetraploid* E. purpurea* polysaccharides in low concentrations promoted the ConA-stimulated lymphocyte secretion of IL-2 and IFN-*γ* more strongly than diploids, and tetraploid* E. purpurea* polysaccharide had an obvious role in promoting the ConA stimulation of lymphocyte secretion of IFN-*γ* compared with the diploids, where the concentration of 0.0312 mg/mL was most obvious. The in vivo results showed that CPE2 and CPE4 at three concentrations could improve lymphocyte proliferation and the immune function of mice induced by cyclophosphamide, and at the same concentration in immunocompromised mice, the lymphocyte proliferation in group CPE4 was better than that in group CPE2. When the polysaccharide concentration was increased, its effect was more obvious. In addition, at the same concentration, the CPE4 group of immune function mice secreted more TNF-*α*, IFN-*γ*, and IL-2 than the CPE2 group. The IL-2 concentration in the spleen lymphocytes of the mice induced by cyclophosphamide was significantly higher than that in the same concentration of the diploid group.

The total polysaccharide content of the medicinal ingredients in tetraploid* E. purpurea* was higher than that in diploid* E. purpurea*. The improved polyploid medicinal plant secondary metabolite contents usually show significantly enhanced biological activity compared with diploids [23, 24]. Studies have revealed that* E. purpurea* crude polysaccharides at 100 *μ*g can significantly stimulate macrophages to kill P815 tumor cells, improve the macrophage production of interleukin level of endothelin 1 (IL-1), and stimulate the proliferation of B lymphocytes in mice. All of these results have indicated that* E. purpurea* crude polysaccharides can enhance humoral immune function [39]. The polysaccharide fraction (M 5000-50000) can stimulate phagocytosis by macrophages and the proliferation of T lymphocytes, improving immune activity [40]. Porcaro et al. (2003) demonstrated that mannose receptor expressed on E‐clone macrophages contributed to phagocytosis of unopsonized microorganisms [41]. Yeast-derived particulate *β*-glucan (p-*β*-glucan) shows it has the ability to activate macrophages and dendritic cells via the dectin-1 pathway [42]. Pectic polysaccharides have certain complement activity that could improve immunity in vivo [43]. Experiments have demonstrated that the TNF-*α* produced after drug-induced rat peritoneal macrophage activation is dependently stimulated by acidic* E. purpurea* in a concentration (3.7 *μ*g/mL-500 *μ*g/mL) that could stimulate the activation of macrophages to secrete IFN-*β*_2_, while the* E. purpurea* acidic arabinogalactan could also dependently stimulate macrophage phagocytosis in the range of 20-200 *μ*g/mL [16, 44]. Given that tetraploid* E. purpurea* polysaccharides achieved their best immune effect at lower concentrations and had stronger immune activities at the same concentration and that the ^1^H-NMR spectrum showed a relative content difference in the partial polysaccharides group between CPE4 and CPE2, we speculated that the tetraploid* E. purpurea *partial polysaccharides group, which had stronger immunological activity similar to pectin polysaccharide or acid arabinogalactan, could have significantly increased content due to the chromosome doubling further to improve the immune activity of* E. purpurea*.

Unfortunately, the present study did not separate the major components of* Echinacea* polysaccharides to identify their content and structural differences. In the future, conducting such studies will be helpful for further investigating and analyzing the influence of ploidy changes on* E. purpurea* polysaccharide synthesis and immune function.

Additionally, evaluation of efficacy and safety of polyploid plants is requisite before they are used as new medicinal plant resources. The previous study reported that no significant difference existed between tetraploid honeysuckle's acute toxicity and diploid honeysuckle's [45]. Similarly, tetraploid and diploid* Scutellaria baicalensis* had good security and clinical medication safety [46]. Existing literatures revealed that* Echinacea* had no obvious toxic action on acute and long-term toxicity and mutagenic and reproductive toxicity of animals [1, 47]. Our research showed that there were no differences between CPE4 and CPE2 in the overall structure and molecular weight. Additionally, Xu et al. (2014) reported that the chemical profiles of the diploid and tetraploid* E. purpurea* plants were similar [19]. That means the chromosome doubling did not lead to the synthesis of new* E. purpurea* compound. Therefore, we speculated that the tetraploid* E. purpurea* is safe and has no significant toxicity as diploid plants.

## Figures and Tables

**Table 1:** Determination of *E. purpurea* polysaccharides (*n* = 3).

Sample	Sample OD_490_	Carbohydrate content of crude polysaccharide (%)	Average content (%)	RSD (%)
CPE2-1	0.253	44.45		
CPE2-2	0.255	44.85	44.65	0.45
CPE2-3	0.254	44.65		
CEP4-1	0.452	84.89		
CPE4-2	0.460	85.52	85.51	1.03
CPE4-3	0.453	85.10		

**Table 2:** Effects of tetraploid and diploid *E. purpurea* on the proliferation of spleen lymphocytes in mice. *∗* indicates stimulation index of lymphocyte proliferation (SI = Group A567/ConA A567).

Groups	0.0039	0.0078	0.0156	0.0312	0.0625	0.1250	0.2500	0.5000
CPE2_-_*A*_*567*_	0.459 ± 0.013^cde^	0.461 ± 0.022^cde^	0.463 ± 0.018^cde^	0.454 ± 0.008^cde^	0.497 ± 0.005^bc^	0.568 ± 0.015^a^	0.541 ± 0.004^a^	0.479 ± 0.007^bcd^
SI	1.133	1.138	1.143	1.201	1.227	1.402	1.335	1.182
CPE4 _-_*A*_*567*_	0.436 ± 0.002^c^	0.495 ± 0.017^b^	0.541 ± 0.005^a^	0.571 ± 0.002^a^	0.468 ± 0.006^b^	0.462 ± 0.010^bc^	0.431 ± 0.004^c^	0.435 ± 0.011^c^
SI	1.076	1.223	1.335	1.409	1.156	1.14	1.064	1.074
ConA	0.405 ± 0.012	0.405 ± 0.012	0.405 ± 0.012	0.405 ± 0.012	0.405 ± 0.012	0.405 ± 0.012	0.405 ± 0.012	0.405 ± 0.012

*∗*a–d: data within a column without the same superscripts differ significantly.

**Table 3:** Effects of tetraploid and diploid *E. purpurea* on the production of IL-2 and IFN-*γ* in mouse lymphocytes.

Group		Content of *E. purpurea *polysaccharide (mg/mL)							
		0.0039	0.0078	0.0156	0.0312	0.0625	0.1250	0.2500	0.5000
IL-2 (pg/mL)	CPE2	688.63 ± 28.01^c^	730.06 ± 43.5^abc^	708.39 ± 31.09^bc^	768.7 ± 14.90^abc^	780.27 ± 20.96^abc^	879.48 ± 8.15^a^	700.00 ± 30.14^bc^	704.66 ± 9.54^bc^
	CPE4	776.78 ± 62.04^abc^	786.39 ± 49.20^abc^	773.28 ± 25.13^abc^	866.99 ± 10.51^ab^	773.98 ± 9.27^abc^	737.73 ± 22.59^abc^	727.83 ± 47.66^abc^	735.69 ± 45.68^abc^
	ConA	533.64 ± 25.07							
IFN-*γ* (pg/mL)	CPE2	1157.43 ± 76.3^b^	1164.37 ± 0.0^b^	1129.65 ± 34.71^bc^	1178.26 ± 51.03^b^	1164.37 ± 97.09^b^	1223.40 ± 31.25^b^	1115.04 ± 50.07^bc^	1119.31 ± 41.66^b^
	CPE4	1187.52 ± 16.02^b^	1247.70 ± 20.83^ab^	1299.79 ± 24.30^ab^	1430.00 ± 44.31^a^	1268.54 ± 90.18^ab^	1251.18 ± 21.23^ab^	1212.98 ± 12.88^b^	1326.41 ± 34.72^b^
	ConA	973.40 ± 17.36							

*∗*  ^a–d^Data within a column without the same superscripts differ significantly.

**Table 4:** Effects of tetraploid and diploid* E. purpurea* on proliferation of spleen lymphocytes in mice immunosuppressed by cyclophosphamide (x ± S, *n* = 7).

Groups	Dose (mg/Kg BW)	*A*_*567*_	SI
C	--	0.358 ± 0.007^f^	--
M	--	0.264 ± 0.004	0.737
E	90	0.672 ± 0.006^cd^	1.877
CPE2-L	40	0.566 ± 0.009^e^	1.581
CPE2-M	80	0.633 ± 0.011^d^	1.768
CPE2-H	160	0.707 ± 0.017^bc^	1.975
CPE4-L	40	0.585 ± 0.008^e^	1.634
CPE4-M	80	0.737 ± 0.018^ab^	2.059
CPE4-H	160	0.756 ± 0.025^a^	2.112

*∗* indicates stimulation index of lymphocyte proliferation (SI = Group *A*_*567*_/C *A*_*567*_).

*∗*  ^a–f^Data within a column without the same superscripts differ significantly.

**Table 5:** Effects of tetraploid and diploid *E. purpurea *on viscera indices of mice immunosuppressed by cyclophosphamide (x ± S, *n* = 7).

Groups	Dose (mg/mL)	Viscera indices (mg/g)				
		Heart	Liver	Spleen	Lung	Kidney
C	--	0.641 ± 0.008^d^	5.391 ± 0.103^b^	0.667 ± 0.018^c^	0.771 ± 0.021^e^	1.587 ± 0.011^ab^
M	--	0.579 ± 0.010^e^	4.969 ± 0.062^d^	0.458 ± 0.016^d^	0.763 ± 0.009^e^	1.469 ± 0.013^e^
E	90	0.822 ± 0.003^b^	5.294 ± 0.062^bc^	0.991 ± 0.069^ab^	0.856 ± 0.010^abc^	1.554 ± 0.020^bcd^
CPE2-L	40	0.720 ± 0.013^c^	4.946 ± 0.058^d^	0.863 ± 0.019^b^	0.799 ± 0.016^de^	1.457 ± 0.007^e^
CPE2-M	80	0.736 ± 0.010^c^	5.008 ± 0.025^d^	0.933 ± 0.060^ab^	0.810 ± 0.016^cde^	1.496 ± 0.019^de^
CPE2-H	160	0.697 ± 0.020^c^	5.133 ± 0.060^cd^	1.055 ± 0.059^ab^	0.815 ± 0.015^bcde^	1.507 ± 0.029^cde^
CPE4-L	40	0.711 ± 0.016^c^	5.374 ± 0.051^b^	0.946 ± 0.051^ab^	0.842 ± 0.009^abcd^	1.574 ± 0.015^b^
CPE4-M	80	0.932 ± 0.009^a^	5.072 ± 0.015^d^	0.968 ± 0.041^ab^	0.865 ± 0.021^ab^	1.561 ± 0.018^bc^
CPE4-H	160	0.957 ± 0.023^a^	5.745 ± 0.081^a^	1.125 ± 0.095^a^	0.874 ± 0.041^a^	1.636 ± 0.024^a^

*∗*: [viscera index = weight of organ (mg)/weight of mouse (g) × 10].

*∗*  ^a–e^Data within a column without the same superscripts differ significantly.

## Abbreviations

CPE2: Crude polysaccharide of diploid* E. purpurea*
CPE4: Crude polysaccharide of tetraploid* E. purpurea*
ConA: Concanavalin A
MTT: 3-(4,5-Dimethylthiazol-2-yl)-2,5-diphenyltetrazolium bromide
IL-2: Interleukin-2
IFN-*γ*: Interferon-*γ*
TNF-*α*: Tumor necrosis factor-*α*
C: Normal control group
M: Model control group
E: Esberitox group
CPE2-L, CPE2-M, and CPE2-H: Diploid* E. purpurea* groups at a low, medium, and high dose, respectively
CPE4-L, CPE4-M, and CPE4-H: Tetraploid* E. purpurea* groups at a low, medium, and high dose, respectively.
